# Supplementary material for: Silmitasertib (CX-4945) Disrupts ERα/HSP90 Interaction and Drives Proteolysis through the Disruption of CK2β Function in Breast Cancer Cells
Source: Cancers (Basel). 2024 Jul 10;16(14):2501. doi: 10.3390/cancers16142501 (PMC11274397; doi:10.3390/cancers16142501)
Supplement: Supplementary file 1 [file cancers-16-02501-s001.zip › cancers-3092821-supplementary.pdf]

Supplementary Materials

Silmitasertib (CX-4945) Disrupts ERα/HSP90 Interaction and Drives Proteolysis through the Disruption of CK2β Function in Breast Cancer Cells

Hogyoung Kim, Emma Elkins, Rahib Islam, Bo Cao, Nour Abbes, Kaela Battles, Sihyoung Kim, Sichan Kim and Christopher Williams

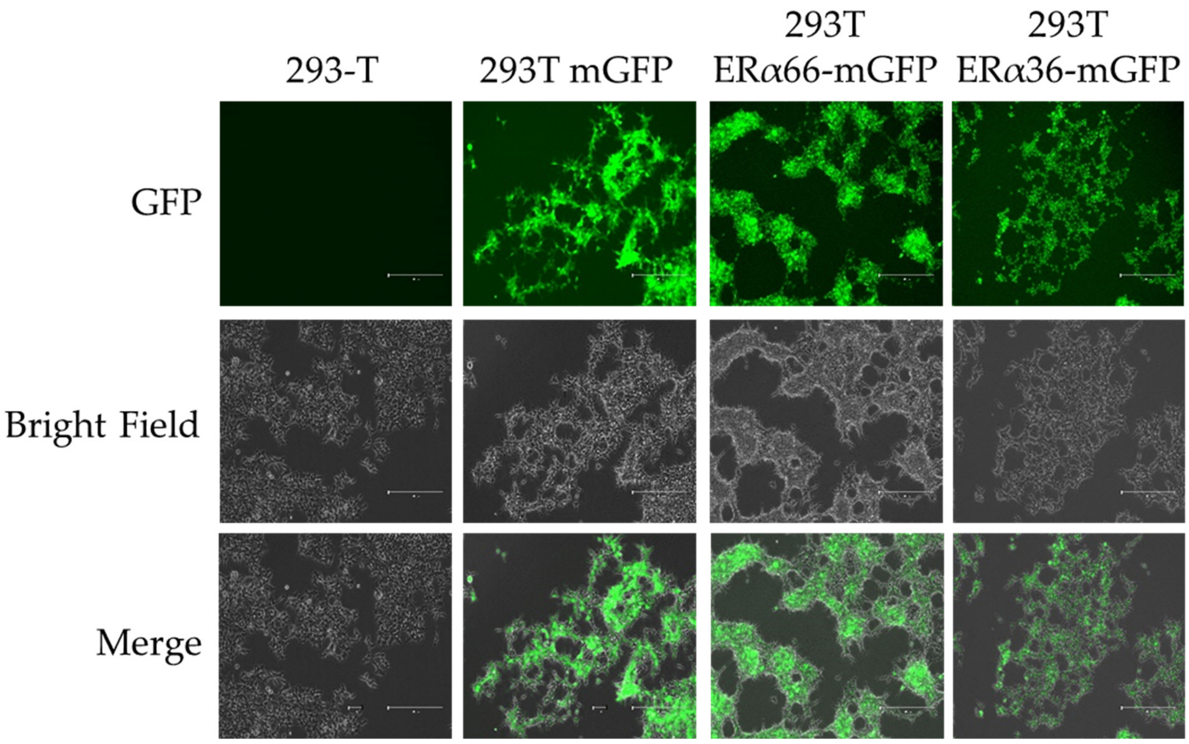

Figure S1. Expression of GFP in 293T cells transfected with mGFP, ERα66-mGFP, or ERα36-mGFP.

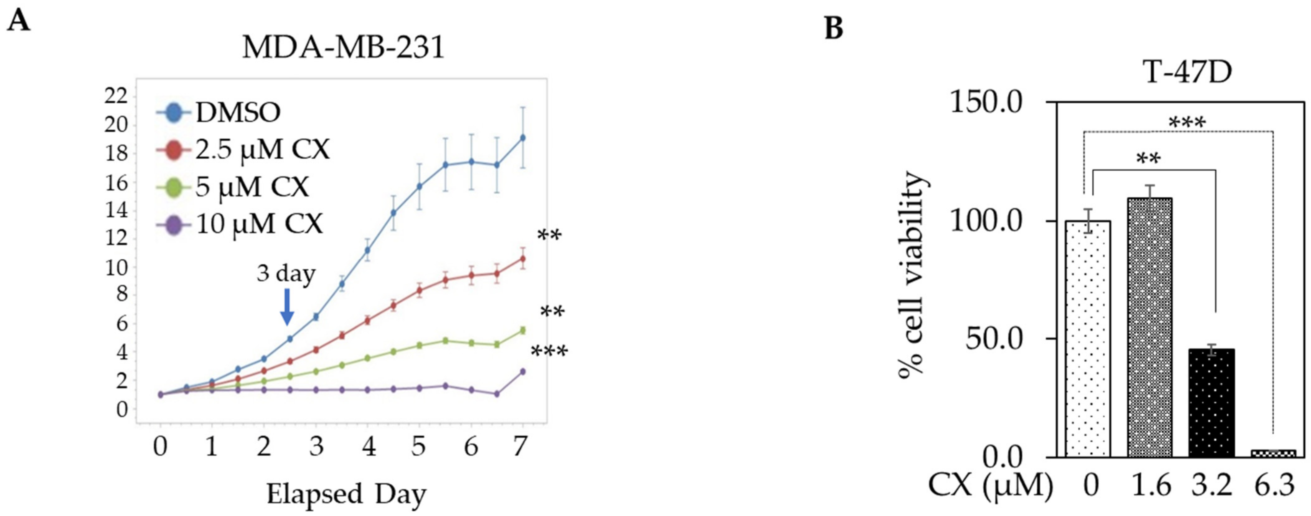

Figure S2. Proliferation analysis of the cell lines. (A) Cell confluence of MDA-MB-231 was measured growing the cells in IncuCyte. Cells were scanned every 12-hour during the times indicated. (B) Proliferation of the T-47D was measured by the Alamar blue assay after 3 day. The data is presented as percent cell confluence ± S.D. Statistically significant results are marked with ** $p \leq 0.01$, *** $p \leq 0.005$, and compared with the control group.

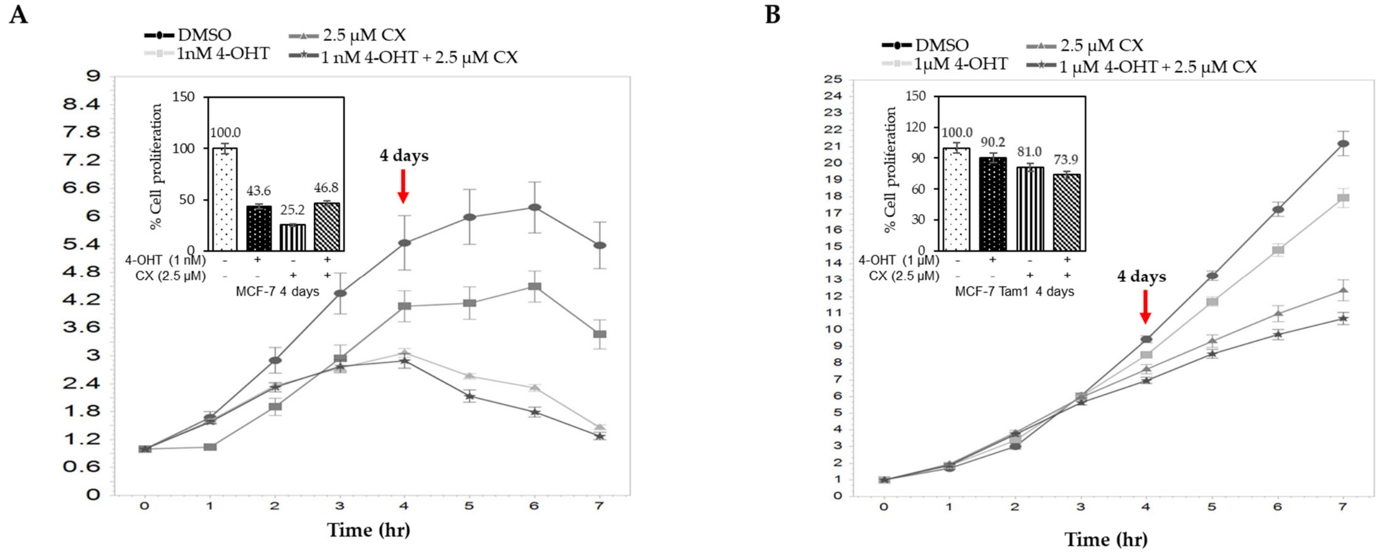

Figure S3. The effects of 4-OHT, CX, and drug combination on cancer cell growth. Live cell imaging of cell confluence with exposure of MCF-7 (A) and MCF-7 Tam1 cells (B) with CX, 4-OHT and their combination. MCF-7 and MCF-7 Tam1 were exposed to single and combination of drugs for up to 7 days. The bar graph was the represented cell proliferation of BCa treated with 4-OHT, CX, or their combination for 4 days (inset). The values shown are the mean ± SD of two independent experiments performed in triplicate. The surviving fraction is the percentage of viable cells in comparison to the control.

Figure 2B

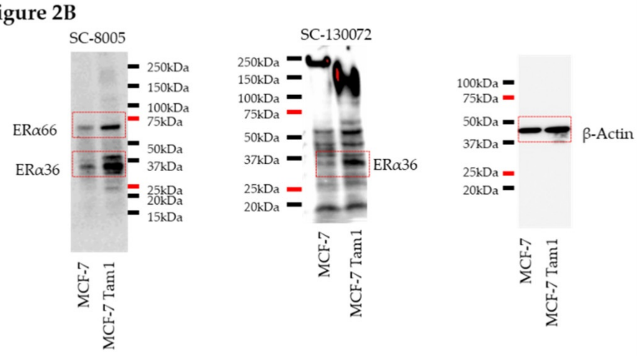

Figure 2C

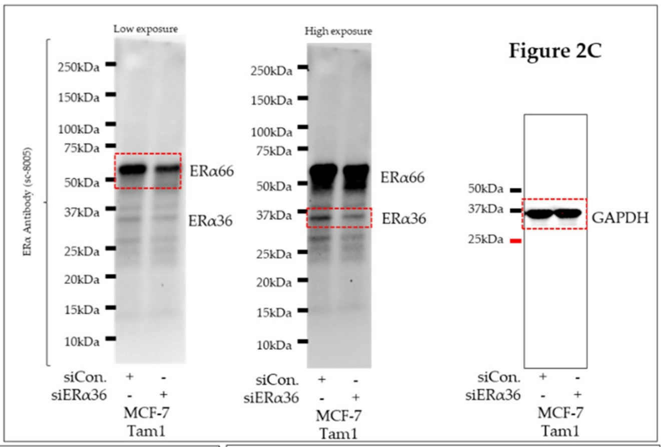

Figure 2E

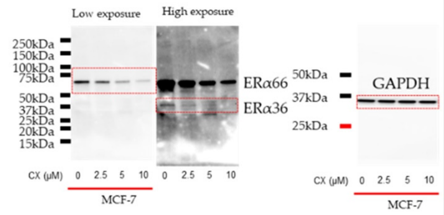

Figure 2F

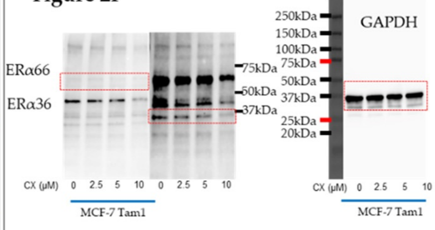

Figure 2 I

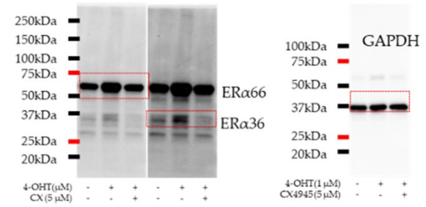

Figure 2G

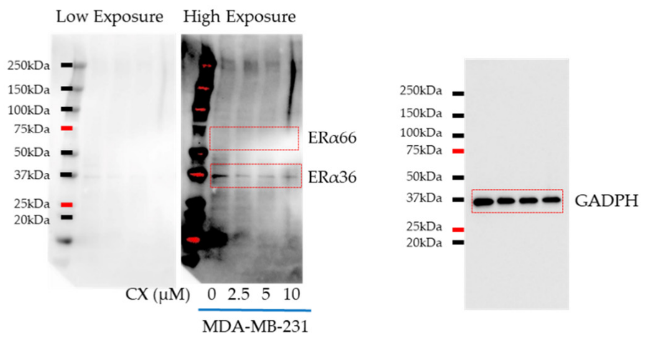

Figure 2H

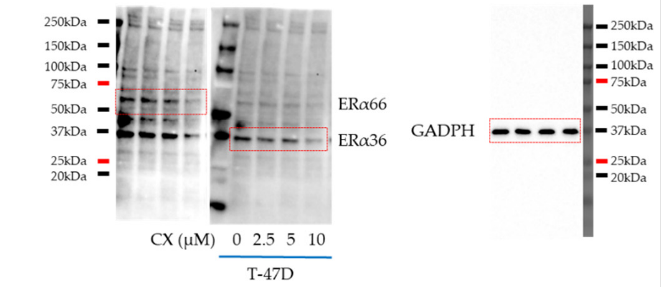

Figure 3A

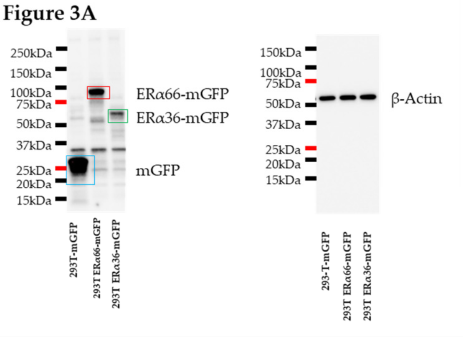

Figure 3B

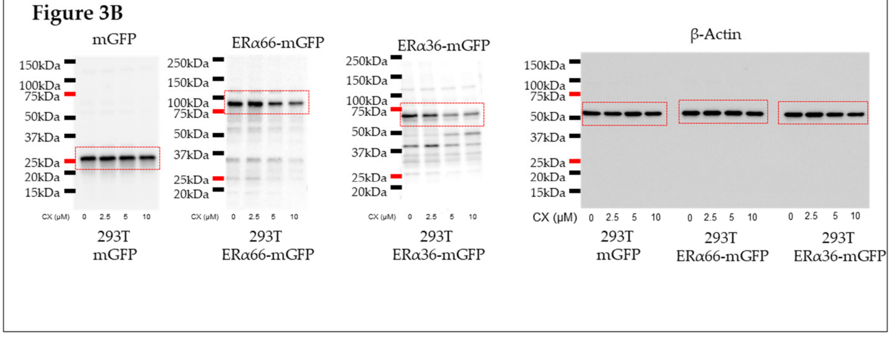

Figure 3C

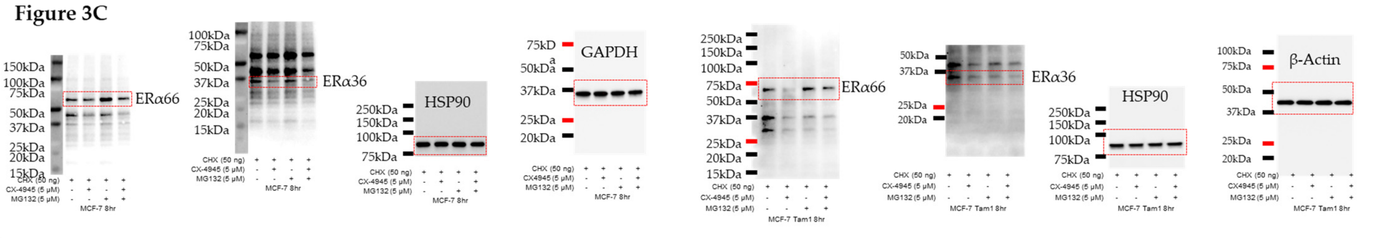

Figure 4B

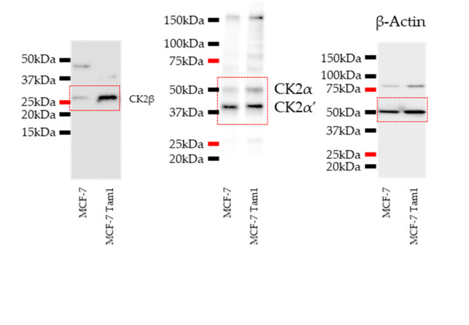

Figure 4C

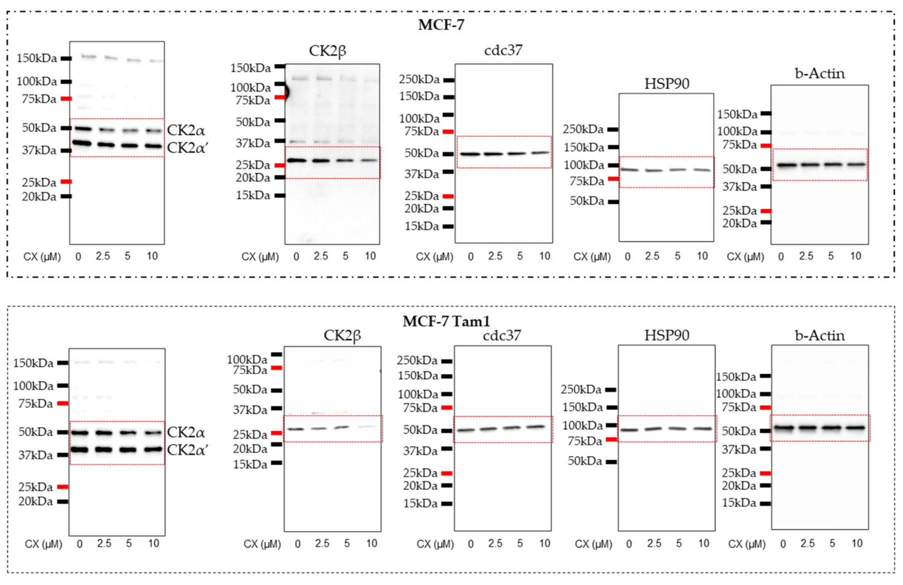

Figure 4E

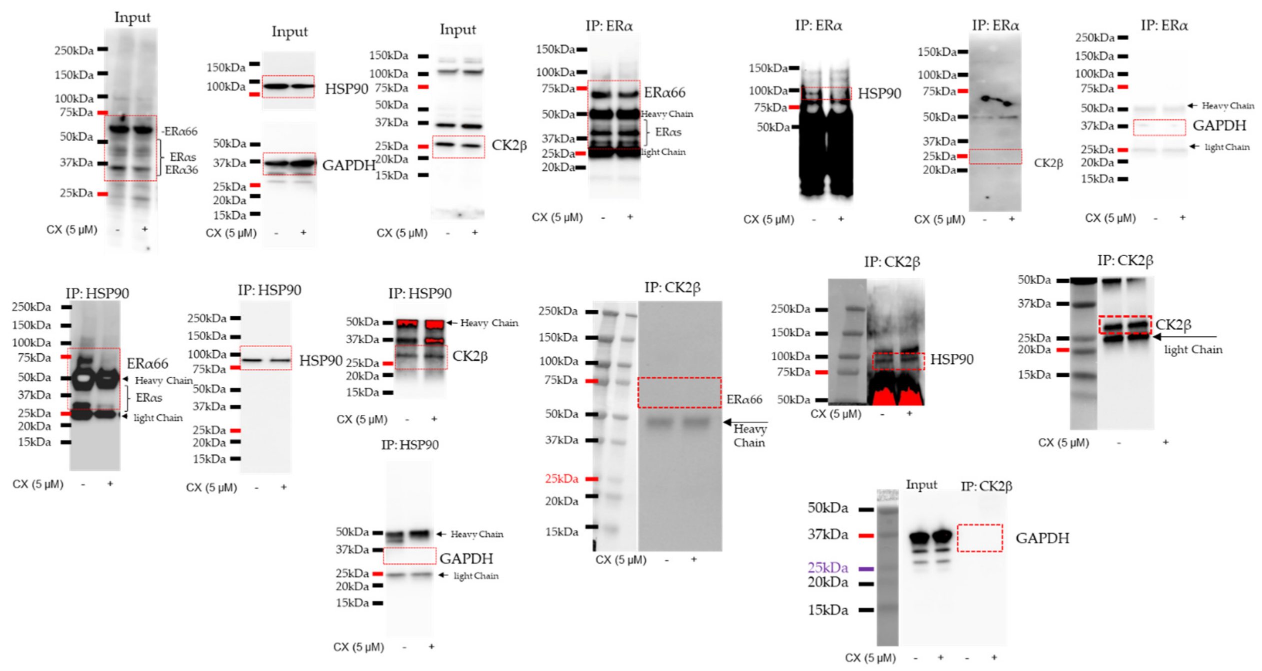

Figure 4G

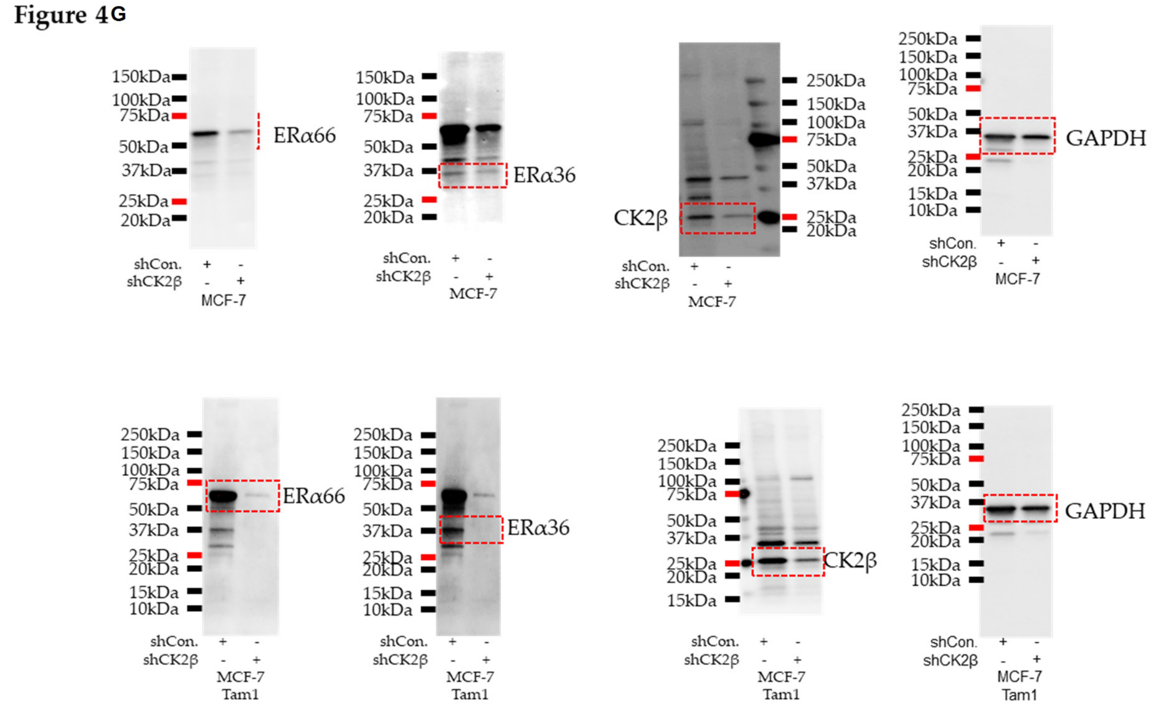

Figure S4. Information of Full blot.

Table S1. Gene, primer orientation, primer sequence (5′ to 3′), and National Center for Biotechnology Information (NCBI) accession number and sequence for primers used in real-time quantitative PCR assays.

Primer Name		Sequence
CSNK2B (CK2 β)	F	TGAGCAGGTCCCTCACTACC
	R	GTAGCGGGCGTGGATCAAT
ERα66	F	GGTGCCCTACTACCTGGAGA
	R	TCTGAATTTGGCCTGTAGAATG
ERα36	F	GACAGGAACCAGGGAAAA
	R	TCTACATGTGAGATACCAGA
GAPDH	F	GACAGTCAGCCGCATCTTCT
	R	TTAAAAGCAGCCCTGGTGAC
